# Cost evaluation of acute ischemic stroke in Latin America: a multicentric study

**DOI:** 10.1016/j.lana.2024.100959

**Published:** 2024-12-06

**Authors:** Luiza Borba Dittrich, Ana Paula Beck da Silva Etges, Joana Siqueira de Souza, Miriam Allein Zago Marcolino, Eva Rocha, Pablo Amaya, Miguel A. Barboza, Andrés Gaye Saavedra, Gonzalo Pérez Hornos, Carlos Abanto, Ana Lucía Castillo-Soto, Natalia Llanos-Leyton, Virginia Pujol Lereis, María Soledad Rodriguez Pérez, Matías Alet, Victor Navia, Solange Lopez, Antonio Arauz, Fabiola Serrano, Bruna Chwal, Leonardo Augusto Carbonera, Raul Gomes Nogueira, Gustavo Saposnik, Carisi Anne Polanczyk, Sheila Cristina Ouriques Martins, Ana Cláudia de Souza

**Affiliations:** a Graduate Program in Industrial Engineering, Universidade Federal do Rio Grande do Sul, Porto Alegre, RS, Brazil; b National Institute of Science and Technology for Health Technology Assessment (IATS) - Brazil, Porto Alegre, Brazil; c Graduate Program in Epidemiology, Universidade Federal do Rio Grande do Sul, Porto Alegre, RS, Brazil; d Department of Neurology, Universidade Federal de São Paulo, São Paulo, Brazil; e Neurology Department, Fundación Valle del Lili, Cali, Colombia; f Neurosciences Department, Hospital Dr. Rafael A. Calderon Guardia, Costa Rica; g Instituto de Neurología, Hospital de Clínicas, Montevideo, Uruguay; h Cerebrovascular Disease Research Center, National Institute of Neurological Sciences, Lima, Peru; i Department of Global Health, University of Washington, Seattle, WA, USA; j Centro de Investigaciones Clínicas, Fundación Valle del Lili, Cali, Colombia; k Centro Integral de Neurología Vascular, Departamento de Neurología, FLENI, Buenos Aires, Argentina; l Neurology Department, Hospital Padre Hurtado, Santiago, Chile; m Administrative Department, Hospital Padre Hurtado, Santiago, Chile; n Stroke Department, Instituto Nacional de Neurología y Neurocirugía Manuel Velasco Suarez, México City, Mexico; o Neurology Department, Hospital de Clínicas de Porto Alegre, Porto Alegre, RS, Brazil; p Neurology and Neurosurgery Department Hospital Moinhos de Vento, Porto Alegre, RS, Brazil; q Neurology and Neurosurgery Department, University of Pittsburgh School of Medicine, Pittsburgh, PA, USA; r Department of Medicine, University of Toronto, Toronto, Ontario, Canada

**Keywords:** Ischemic stroke, Cost, Cost evaluation, Cost impact

## Abstract

**Background:**

Current literature highlights a gap in precise stroke cost data for Latin America. This study measures the real costs associated with acute ischemic stroke care in Latin America using Time–Driven Activity-Based Costing (TDABC). The findings aim to lay a solid foundation for adopting value-based healthcare (VBHC) strategies in the region.

**Methods:**

The study is an observational, multicenter, international analysis of direct costs and outcomes for patients hospitalised with acute ischemic stroke from December 2021 to December 2022. Data from stroke centres in Argentina, Brazil, Chile, Colombia, Costa Rica, Mexico, Peru, and Uruguay were analysed. Costs were stratified by country. Factors such as favourable outcomes based on the modified Rankin Scale (mRS 0–2), clinical risk levels, and treatment interventions were considered for the analysis. Generalized Estimating Equation (GEE) models were utilised to assess the relationship of clinical variables with the total cost per patient.

**Findings:**

A total of 1106 patients were included in the study. Among these patients, 74% received medical treatment alone, 18% received intravenous thrombolysis (IVT), 4% underwent mechanical thrombectomy (MT), and 3% received combined IVT plus MT. The mean cost per patient was I$ 12,203 (SD I$ 15,055), with 49% achieving a favourable functional outcome. Compared to medical treatment alone, MT incurred costs 3.1 times higher, with an incremental cost of I$ 20,418 per patient (p < 0.0001). Across all countries, costs increased according to patients' clinical risk and treatment options, with length of hospital stay emerging as the primary cost driver.

**Interpretation:**

Our study highlights significant disparities in stroke costs across healthcare services in Latin America, influenced by variations in treatment accessibility, patient outcomes, and clinical risk profiles. These findings offer essential insights for shaping health policy decisions to enhance the long-term sustainability of stroke care in the region.

**Funding:**

The project received funding from the World Stroke Organization and 10.13039/100001003Boehringer Ingelheim (BI) IS 0135-0352.

**Research in context**

**Evidence before this study**

A systematic literature search was conducted on July 16, 2021, across PubMed, EMBASE, LILACS, and SciELO to identify studies assessing the costs and outcomes of acute ischemic stroke treatment in Latin America. The following search terms were used: (“stroke” OR “acute ischemic stroke” OR “ischemic stroke”) AND (“treatment” OR “acute treatment”) AND (“costs” OR “cost evaluation” OR “cost analysis” OR “economic evaluation” OR “cost-effectiveness analysis” OR “cost-effectiveness”) AND (“Latin America” OR “Latin American countries” OR specific country names in the region). This search identified 589 citations, including 206 from PubMed, 335 from EMBASE, 28 from LILACS, and 20 from SciELO, with 491 non-duplicate articles screened. After applying inclusion criteria—studies focusing on economic assessments of acute ischemic stroke treatment performed in Latin America—18 articles were retrieved and included for review. The search revealed that while some studies provided insights into stroke costs, the literature lacked comprehensive and standardised analyses using robust methodologies like Time–Driven Activity-Based Costing (TDABC). This gap highlighted the need for detailed evaluations to inform healthcare policy and improve resource allocation for stroke care in the region.

**Added value of this study**

This study is the first to use TDABC to evaluate the direct costs of acute ischemic stroke treatment across eight Latin American countries. By integrating data from public and private stroke centres, the study provides robust evidence on cost variability across treatment modalities and clinical risk profiles. The findings also quantify the economic impact of different treatment approaches, including medical management, intravenous thrombolysis, and mechanical thrombectomy, addressing a critical knowledge gap in regional stroke economics and establishing a foundation for implementing value-based healthcare in Latin America.

**Implications of all the available evidence**

The evidence presented underscores the urgent need for tailored health policy interventions aimed at reducing the economic burden of stroke care in Latin America. The significant variabilities in treatment costs and outcomes across countries call for a concerted effort to improve treatment accessibility and optimize resource allocation. Policymakers can leverage these insights to enhance the efficiency and sustainability of stroke care systems, ultimately aiming to improve patient outcomes and achieve better value for healthcare expenditures. This study’s methodology and findings could serve as a model for other regions facing similar healthcare challenges.

## Introduction

Stroke poses a significant health challenge globally, ranking among the leading causes of death and disability worldwide.^1^, ^2^, ^3^ This burden is particularly pronounced in low- and middle-income countries (LMICs) compared to high-income countries (HICs), where limited healthcare access exacerbates the impact.^4^ Projections indicate a concerning 50% increase in the number of stroke deaths from 2020 to 2050 in LMICs, with costs estimated to rise from US$891 billion annually to US$2.31 trillion by 2050.^5^

Despite the well-documented benefits and cost-effectiveness of interventions such as intravenous thrombolysis (IVT) and mechanical thrombectomy (MT), access remains limited, with less than 2% of stroke patients having access in resource-constrained settings.^5^, ^6^, ^7^, ^8^, ^9^, ^10^ Disparities in awareness, healthcare access, and infrastructure between LMICs and HICs present significant obstacles to the widespread adoption of these treatments.^10^, ^11^, ^12^ Local government support plays a pivotal role, as stroke networks rely heavily on budget allocations for infrastructure, healthcare personnel, and the implementation of effective, sustainable programs guided by national policies.^12^

A literature review indicates a lack of standardised methods for measuring, estimating, and reporting stroke costs.^13^ In pursuit of more effective, patient-centred, and data-driven care pathways, value-based healthcare (VBHC) has emerged as a promising strategy worldwide.^14^, ^15^, ^16^ Among the prerequisites for implementing and managing care services based on value is the accurate measurement of outcomes and costs. Time–Driven Activity-Based Costing (TDABC) emerges as the gold standard, offering a practical framework for optimising healthcare resource allocation and enhancing stroke care delivery.^16^, ^17^, ^18^, ^19^

This multicenter study aims to measure stroke costs in Latin American countries, utilising VBHC strategies and TDABC to establish more effective care pathways and enhance resource allocation regionally.

## Methods

### Study design

A cross-sectional, multicenter international study was undertaken to assess costs and outcomes, drawing patient data from a single designated stroke centre in each of the following countries: Argentina, Brazil, Chile, Colombia, Costa Rica, Mexico, Peru, and Uruguay. Data collection commenced with a Pilot Phase spanning from June to December 2021, followed by a validation phase, and extended into the Main Phase from June to December 2022. The study adopted a hospital-centric perspective, encompassing both public (n = 6) and private institutions (n = 2). It employed a methodology integrating microcosting and patient-centred approaches, combining retrospective (Pilot Phase) and prospective (Main Phase) data collection methods. This study was conducted following the Consolidated Health Economic Evaluation Reporting Standards (CHEERS) guidelines to ensure transparency and consistency in reporting.

### Participant criteria and hospital selection

Data collection was centred on hospitalized patients aged 18 years and older diagnosed with acute ischemic stroke during the specified timeframe. Centres were chosen based on convenience, considering factors such as the availability of cost data and robust research infrastructure. Eight stroke centres, certified by the World Stroke Organization (WSO) in the mentioned countries, were selected for their proficiency in stroke care, treating a minimum of 100 stroke patients annually, and possessing clinical research experience, ensuring high-quality data for the current study.

### Ethical considerations

Ethical approval was obtained from the coordinating centre, Hospital Moinhos de Vento in Brazil (CAAE: 53723521.8.0000.5330), and the Research Ethics Committees in all participating countries approved the study. Since data acquisition was performed via medical records under hospital protocol, informed consent was waived. Patient confidentiality was safeguarded using numerical coding techniques. Each participating centre omitted sensitive data, such as names and personal identifiers, and assigned unique numerical codes to participants before submitting the information to the coordinating centre. The data was stored in encrypted databases with restricted access, and all personnel were trained in confidentiality and data protection.

### Research conduction

The study comprised the Pilot and the Main phase (Supplementary Material I). During the Pilot phase, data were retrospectively collected from these certified hospitals, with 30 consecutive patients per centre. The primary aim of this phase was to evaluate the feasibility of data collection and cost analysis in the hospitals. Subsequently, upon successfully validating the pilot phase data (n = 208), the main phase expanded data collection to an additional 898 patients from these centres, totalling 1106 patients across eight centres from different countries. In this phase, all acute ischemic stroke patients prospectively admitted to the hospitals from June to December 2022 were included. The research coordinating centre provided initial 1-h online training sessions to the financial departments of each centre to ensure accurate data on infrastructure costs, salaries, medications, and devices before the study began. Following this, investigators were trained to collect precise data on professional time per patient, number of exams during hospitalisation, medications, and procedures. If there were any doubts or difficulties, additional meetings were conducted. The coordination team also monitored the quality of cost and effectiveness data through frequent remote monitoring meetings.

### Measurement of costs and healthcare outcomes

The methodology systematically evaluated the direct costs associated with acute ischemic stroke treatment across the entire care pathway, employing the TDABC method (Supplementary Material II).^16^ This method estimated costs using two primary data: the cost capacity rate (CCR in $/unit of time) per resource and the estimated length of time each patient consumed from each resource, in each activity, over its care cycle. Following this methodology, we mapped out the care process and identified the main activities undergone by each patient. We listed all resources and departments involved, estimated the total expenses for each resource, and calculated the hourly capacity and unit cost rate for each resource or department. Then, we analysed the time devoted to each patient by every resource, developed equations for time and costs, calculated the cost per patient, and performed further analyses. In addition to the basic demographic information and stroke subtypes through the TOAST classification (Trial of ORG 10172 in Acute Stroke Treatment), validated stroke scores were used to evaluate healthcare status, including the modified Rankin Score (mRS) at discharge and 3 months and the National Institute for Health Stroke Scale (NIHSS) at admission, in addition to mortality and hospital length of stay.

#### Mapping of care flow and identification of hospital resources

The study team mapped the care flow based on the hospital's ischemic stroke care routines and protocols, validated by a vascular neurologist. It was assumed that patient flow was similar across all eight centres due to their accreditation by the WSO. Five macrophases were identified across the care pathway: emergency, general ward, angiography, intensive care unit (ICU), and stroke unit. The resources consumed in these phases included professionals' salaries, the physical structure of each department, exams, procedures the patient underwent, medications prescribed, and IVT medications and/or materials used for performing MT, considering the costs of resources available at each centre (Supplementary Material II).

#### Estimating total costs of hospital resources

For each identified resource, total mean costs were estimated. Labour costs were calculated based on the monthly salary of each professional plus their benefits, considering the volume of consultations across the identified phases. Total costs of hospital infrastructure refer to the fixed monthly costs of the space, such as equipment depreciation, electricity, fees, and other expenses of each department. Data acquisition involved reviewing financial reports from hospital departments. Medication and exam costs are unit costs multiplied by the volume prescribed or performed for each patient, with individual consumption extracted from reviewing medical records and prescriptions. Exams were computed based on the identification in the clinical records. The cost of the thrombolytic agent is estimated by the unit cost of alteplase or tenecteplase and the dosing administered (cost per milligram). The costs of the MT devices refer to the sum of materials costs used per patient during the procedure.

#### Estimating hourly capacity and unit cost rates

The capacity of each department's space was estimated based on room and bed availability, while the contracted workload determined labour capacity for one month. To account for inherent process losses, 85% of the installed capacity was considered for both resources. Each resource's cost-capacity rate (CCR) can be calculated using the cost per resource and capacity information.

#### Time allocation per patient and cost equations

The average time for each patient consultation was estimated through employee allocation scales, interviews with professionals, and hospital guidelines. Interviews with department managers and hospital productivity reports determined the time spent in each department per patient. Detailed information on each centre's departmental length of stay can be found in Supplementary Material III.

#### Calculating the cost per patient

The time spent on each resource was multiplied by its respective CCR to calculate the individual cost per patient. Subsequently, this value was added to the individual cost of medications, exams, and treatments. Specific clinical outcomes, including the mRS and case mix variables such as NIHSS scores and age, were also selected for collection alongside cost data to enable clinical translation. A standardized spreadsheet (Supplementary Material IV) was created for the data collection, allowing the centres to gather patient-level information consistently, ensuring the reliability of the results.

### Data estimation

Challenges encountered in post-treatment patient communication, coupled with incomplete financial data in specific centres, necessitated a flexible approach to data estimation. Protocols were devised to address the 1.6% of missing information, ensuring maximal patient inclusion. In the case of clinical data, missing values for NIHSS and mRS scores were extrapolated based on admission and discharge data. Missing NIHSS data at admission were imputed using the NIHSS scores recorded at discharge for the same patients, while missing discharge NIHSS data were imputed using the admission scores. For the missing 3-month mRS data, the mRS scores obtained at discharge were used for the corresponding patients. Financial estimates were derived from the collected sample. For the missing cost data, values were extrapolated using the mean values from patients with complete data from each hospital. For centres in Brazil and Chile, data from previous real-world studies were utilized.^20^^,^^21^ Specifically, for the centres in Brazil and Chile, the studies provided comprehensive insights into the cost dynamics of stroke treatment, enhancing the accuracy of our estimations. Noteworthy adaptations were made for medication details in Chile and Costa Rica centres. The medication costs in the Chilean centre were allocated based on the proportion of each medication's usage at the centre and distributed proportionally to each patient's length of hospital stay. No data on medication usage at the centre in Costa Rica were available, and they were not included in the analysis. The Chilean centre was only a primary stroke centre, and patients undergoing MT were referred to another hospital. Given the complexity of obtaining precise costs for this procedure and considering that MT represented a small proportion of cases in that centre (2%, n = 3), mean values from the referenced studies were employed to approximate these costs.

### Risk stratification

Based on the model validated in a previous Brazilian study^21^ and utilising patient age and NIHSS levels upon hospital admission, the stratification by risk level per treatment was employed to address patient clustering among institutions. This model, previously validated by the Generalized Estimating Equation (GEE) model, categorised patients into low, medium, and high-risk levels. The model confirmed the validity of both the individual and combined effects of NIHSS and age in stratifying risk levels, demonstrating significant associations with favourable outcomes (mRS 0–2) using both univariate and multivariate GEE models. The NIHSS classification criteria and the age cut-off were based on previous studies.^22^ Specifically, the low-risk level included patients under 70 years with an NIHSS score <8, the medium-risk level encompassed patients under 70 years with an NIHSS score between 8 and 15, as well as those older than 70 with an NIHSS score <8, and the high-risk level included patients older than 70 years with an NIHSS score >8 and those under 70 years with an NIHSS score >15.^21^

### Data analysis

Descriptive cost analyses were conducted to present the results, including mean, standard deviation (SD), median, and interquartile intervals (IQR), considering patients' risk levels and treatment modalities (medical treatment, IVT, MT, and combined IVT plus MT). Purchasing Power Parity and conversion to International Dollars (I$) facilitated comparisons across different countries. Clinical outcomes such as mortality rates, mRS scores at hospital discharge and 3 months post-discharge, and hospitalisation length of stay were also compared across treatments, countries, and risk levels. The consolidated sample data was organised in a Microsoft® Excel spreadsheet. Descriptive analyses were performed, with additional statistical analysis conducted in R to examine potential differences in costs per treatment relative to medical treatment. Generalized Estimating Equation (GEE) models were utilised, with Country as the clustering variable, applying a Gamma distribution and Logarithm link function, and employing an Exchangeable correlation structure to assess the relationship of clinical variables (age, sex, mRS at discharge, hospitalisation length of stay, and risk stratification) with the total cost per patient. Post-hoc pairwise comparisons were made using the Tukey method for multiple comparison adjustments, with a significance level of 0.05 for all analyses.

### Role of the funding source

The entities that supported the project did not play any role in the design, analysis, or interpretation of the results of this study.

## Results

### Hospital characteristics

The study included 8 hospitals, 6 of which were academic public institutions and 2 of which were academic private hospitals. Included public hospitals were in Brazil, Mexico, Costa Rica, Uruguay, Peru, Chile; and private hospitals were in Argentina and Colombia. The hospitals varied in capacity, with the total number of beds as follows: Argentina - 113, Mexico - 126, Peru - 135, Uruguay - 350, Chile - 371, Costa Rica - 645, Colombia - 731, and Brazil - 784. Among these hospitals, 6 were comprehensive stroke centres equipped to perform MT, while the remaining 2 were primary stroke centres providing IVT only, located in Chile and Peru.

### Patient characteristics

A total of 1106 patients were included in the study. Most patients, 819 (74%), received medical treatment alone, followed by 203 (18%) who received IVT, 49 (4%) who underwent MT, and 35 (3%) who received IVT followed by MT. The median age was 68 years (IQR 59–77), with 54% male. Upon arrival at the hospital, most patients (38%) were classified as medium risk. The median NIHSS score at hospital admission was 7 (IQR 3–13), and the median mRS score was 2 (IQR 1–4) after 3 months post-discharge. The cause of stroke was undetermined in 33% of cases. The rate of favourable functional outcomes at discharge (mRS 0–2) was 15% in high-risk cases, 55% in moderate-risk cases, and 77% for low-risk patients. The mortality at 3 months was 9% (n = 97). Table 1 provides detailed baseline characteristics of the study sample.

**Table 1:** Baseline characteristics and main outcomes of patients with acute ischemic stroke.

	Global	Argentina	Brazil	Chile	Colombia	Costa Rica	Mexico	Peru	Uruguay
Total patients	1106 (100%)	148 (13%)	157 (14%)	126 (11%)	153 (14%)	197 (18%)	103 (9%)	119 (11%)	103 (9%)
Female	508 (46%)	71 (48%)	78 (50%)	60 (48%)	75 (49%)	81 (41%)	45 (44%)	51 (43%)	47 (46%)
Mean age	67 (14)	70 (14)	66 (12)	67 (13)	72 (14)	66 (15)	61 (17)	65 (15)	66 (14)
NIHSS (Discharge)									
0–7	745 (67%)	136 (92%)	117 (75)	98 (78%)	104 (68%)	109 (55%)	47 (46%)	65 (55%)	69 (67%)
8–15	327 (21%)	10 (7%)	31 (20%)	20 (16%)	37 (24%)	51 (26%)	31 (30%)	11 (11%)	11 (11%)
≥15	124 (11%)	2 (1%)	9 (6%)	8 (6%)	12 (8%)	37 (19%)	25 (24%)	23 (22%)	23 (22%)
Stroke subtype									
Cardioembolic	266 (24%)	25 (17%)	53 (34%)	9 (7%)	49 (32%)	34 (17%)	38 (37%)	25 (21%)	33 (32%)
Large vessel atherosclerosis	236 (21%)	15 (10%)	48 (31%)	42 (33%)	14 (9%)	58 (29%)	14 (14%)	33 (28%)	12 (12%)
Lacunar	138 (12%)	16 (11%)	13 (8%)	32 (25%)	10 (7%)	22 (11%)	10 (10%)	15 (13%)	20 (19%)
Other etiology	105 (9%)	19 (13%)	17 (11%)	3 (2%)	11 (7%)	28 (14%)	14 (14%)	8 (7%)	5 (5%)
Undetermined	361 (33%)	73 (49%)	26 (17%)	40 (32%)	69 (45%)	55 (28%)	27 (26%)	38 (32%)	33 (32%)
mRS 3 months									
0	269 (24%)	67 (45%)	18 (11%)	29 (23%)	37 (24%)	64 (32%)	12 (12%)	4 (3%)	38 (37%)
1	192 (17%)	26 (18%)	28 (18%)	29 (23%)	34 (22%)	28 (14%)	23 (22%)	12 (10%)	12 (12%)
2	165 (15%)	24 (16%)	36 (23%)	11 (9%)	24 (16%)	38 (19%)	17 (17%)	9 (8%)	6 (6%)
3	162 (15%)	17 (11%)	27 (17%)	33 (26%)	13 (8%)	25 (13%)	14 (14%)	24 (20%)	9 (9%)
4	150 (14%)	9 (6%)	30 (19%)	14 (11%)	14 (9%)	14 (7%)	15 (15%)	46 (39%)	8 (8%)
5	71 (6%)	1 (1%)	13 (8%)	5 (4%)	10 (7%)	11 (6%)	12 (12%)	17 (14%)	2 (2%)
6	97 (9%)	4 (3%)	5 (3%)	5 (4%)	21 (14%)	17 (9%)	10 (10%)	7 (6%)	28 (27%)
Treatments									
Medical treatment	819 (74%)	121 (82%)	99 (63%)	100 (79%)	104 (68%)	148 (75%)	71 (69%)	108 (91%)	67 (65%)
IVT	203 (18%)	13 (9%)	45 (29%)	23 (18%)	23 (15%)	35 (17%)	28 (27%)	11 (9%)	28 (27%)
MT	49 (4%)	8 (5%)	10 (6%)	3 (2%)	16 (10%)	5 (3%)	3 (3%)	NA	4 (4%)
IVT + MT	35 (3%)	6 (4%)	3 (2%)	NA	10 (7%)	11 (6%)	1 (1%)	NA	4 (4%)
Risk levels									
High risk	351 (32%)	14 (9%)	40 (25%)	30 (24%)	61 (40%)	88 (45%)	47 (46%)	40 (34%)	31 (30%)
Medium risk	422 (38%)	85 (57%)	67 (43%)	36 (29%)	54 (35%)	68 (35%)	27 (26%)	51 (43%)	34 (33%)
Low risk	333 (30%)	49 (33%)	50 (32%)	60 (48%)	38 (25%)	41 (21%)	29 (28%)	28 (24%)	38 (37%)

IVT = Intravenous Trombolysis; MT = Mechanical Thrombectomy; mRS = modified Rankin Score; SD = Standard Deviation; NA = Not applicable.

### Direct costs of acute ischemic stroke treatment

The mean cost per patient was I$ 12,203 (SD I$ 15,055). Treatment modalities (p < 0.0001), mRS at discharge (p < 0.0001), risk levels (p < 0.0001), patient age (p = 0.0185), and hospital length of stay (p < 0.0001) significantly influenced cost variability, while gender showed no significant impact (p = 0.375). IVT incurred an average incremental cost of I$ 5195 per patient (p < 0.0001) compared to medical treatment alone, while MT incurred I$ 20,418 per patient (p < 0.0001) and combined IVT plus MT incurred I$ 19,285 (p < 0.0001). MT and IVT plus MT incurred higher costs than IVT alone (p < 0.0001), as shown in Table 2.

**Table 2:** Direct costs associated with treatment modality, mRS scores at discharge, risk levels, and stroke subtypes.

	General (SD)	Medical treatment	IVT	MT	IVT + MT
Mean cost per patient	I$ 12,203 (I$ 15,055)	I$ 9735	I$ 14,930	I$ 30,153	I$ 29,019
Labour costs	I$ 4699 (I$ 8573)	I$ 3969	I$ 5741	I$ 11,049	I$ 5907
Structure costs	I$ 5014 (I$ 6272)	I$ 4520	I$ 5733	I$ 9290	I$ 6407
Exams	I$ 1130 (I$ 1272)	I$ 1037	I$ 1093	I$ 1558	I$ 2939
Medicines	I$ 448 (I$ 1883)	I$ 255	I$ 715	I$ 599	I$ 3670
Thrombolysis	I$ 1912 (I$ 1510)	–	I$ 1765	–	I$ 2764
Materials thrombectomy	I$ 8234 (I$ 2937)	–	–	I$ 7718	I$ 8487
Mean cost per mRS scale (N)					
0 (189; 17%)	I$ 6633 (I$ 5214)	I$ 5464	I$ 9330	I$ 16,353	I$ 18,698
1 (211; 19%)	I$ 9621 (I$ 10,143)	I$ 7622	I$ 12,648	I$ 19,103	I$ 22,592
2 (143; 13%)	I$ 11,892 (I$ 13,538)	I$ 9384	I$ 20,342	I$ 17,120	I$ 16,999
3 (178; 16%)	I$ 13,518 (I$ 13,491)	I$ 11,314	I$ 16,667	I$ 20,679	I$ 39,248
4 (177; 16%)	I$ 12,900 (I$ 15,115)	I$ 10,792	I$ 13,257	I$ 28,292	I$ 37,796
5 (137; 12%)	I$ 22,008 (I$ 26,895)	I$ 15,908	I$ 25,957	I$ 63,842	I$ 38,307
6 (71; 6%)	I$ 11,377 (I$ 9267)	I$ 10,103	I$ 9779	I$ 18,667	I$ 24,491
Mean cost per mRS category (N)					
0–2 (543; 49%)	I$ 9179 (I$ 10,079)	I$ 7305	I$ 13,374	I$ 17,495	I$ 20,689
3–4 (355; 32%)	I$ 13,210 (I$ 14,307)	I$ 11,051	I$ 15,058	I$ 24,232	I$ 38,466
5–6 (208; 19%)	I$ 18,379 (I$ 23,017)	I$ 14,238	I$ 18,407	I$ 44,821	I$ 32,781
Risk levels					
High risk	I$ 17,438 (I$ 19,781)	I$ 13,552	I$ 17,323	I$ 34,870	I$ 31,117
Medium risk	I$ 9926 (I$ 12,739)	I$ 8342	I$ 12,984	I$ 23,795	I$ 23,310
Low risk	I$ 9572 (I$ 9673)	I$ 8456	I$ 13,477	I$ 18,621	I$ 30,070
Stroke subtypes					
Cardioembolic	I$ 15,492 (I$ 19,360)	I$ 11,312	I$ 17,600	I$ 36,026	I$ 33,694
Large vessel atherosclerosis	I$ 13,565 (I$ 15,083)	I$ 11,809	I$ 18,313	I$ 21,810	I$ 26,421
Lacunar	I$ 8165 (I$ 7753)	I$ 7120	I$ 12,296	I$ 13,958	I$ 18,933
Other etiology	I$ 14,860 (I$ 17,644)	I$ 10,843	I$ 18,014	I$ 41,602	I$ 41,224
Undetermined	I$ 9661 (I$ 11,596)	I$ 8074	I$ 10,693	I$ 25,834	I$ 22,976

IVT = Intravenous Trombolysis; MT = Mechanical Thrombectomy; mRS = modified Rankin Score; SD = Standard Deviation.

Patients with an mRS score of 5 at discharge incurred the highest cost (I$ 22,008), followed by those with an mRS score of 3 (I$ 13,518), while the mRS 0 category had the lowest mean cost (I$ 6633). Patients with mRS scores of 2 or higher had higher costs than those with mRS 0 (p < 0.0001). Additionally, mRS scores of 4, 5 (p < 0.0001) or 6 (p = 0.037) incurred higher costs than mRS 1, with mRS 5 demonstrating higher costs than all other mRS categories (p < 0.0001). The rate of favourable functional outcomes at discharge (mRS 0–2) was 49%, yielding lower costs than mRS 3–4 (p = 0.0067) and mRS 5–6 (p < 0.0001). Moreover, mRS scores of 3–4 presented lower costs than mRS 5–6 (p = 0.0143).

**Fig. 1:**
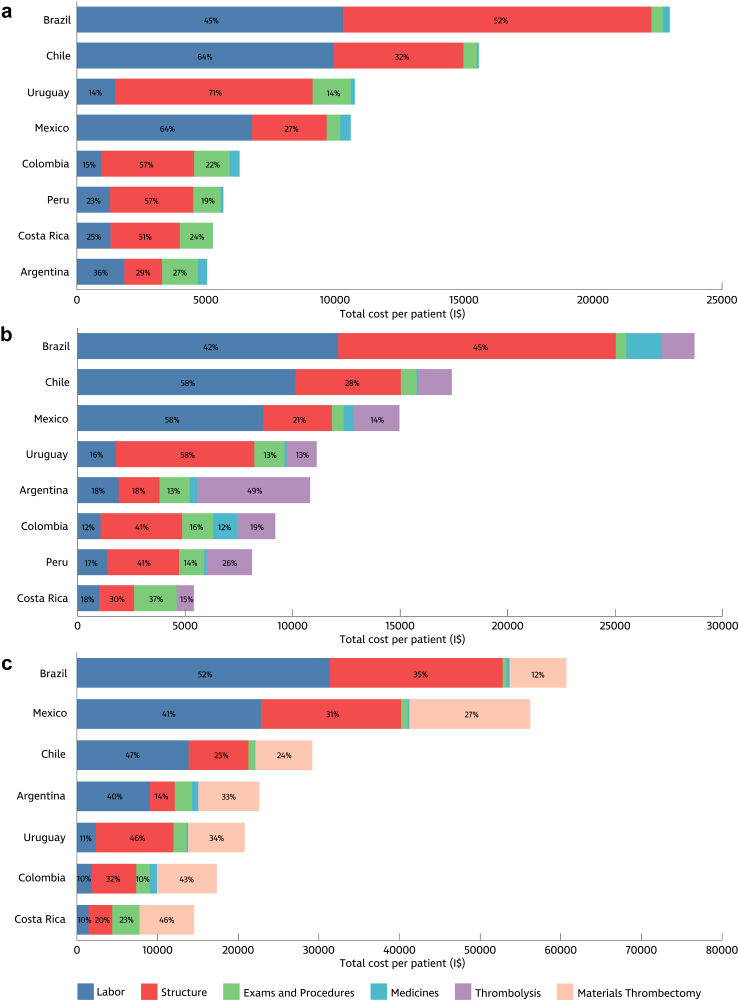
**Cost distribution across treatment types by country. (a)** Medical treatment; **(b)** Intravenous thrombolysis; **(c)** Mechanical thrombectomy. The contribution of each cost item to the total cost is expressed in percentages. Legends with values below 10% were omitted. I$: International Dollars.

Expanding on our examination of the direct costs associated with acute ischemic stroke treatment, it becomes increasingly apparent that a multitude of factors contribute to the overall financial burden. The most expensive costs identified were hospital structure and labour costs, notably driven by the heightened utilisation of departments such as angiography and the Intensive Care Unit (ICU) for patients undergoing MT (Fig. 1). Compared to other departments, these departments entail higher unit cost rates for infrastructure, attributed to increased depreciation costs and expenses for medical supplies, mechanical ventilation, cardiac monitoring, and renal substitutive therapies, and reflect higher labour costs than the general ward. Moreover, high-risk patients, as well as those receiving MT and combined treatments, required prolonged hospitalisations and more expressive medication use, consequently accounting for the highest mean costs.

### Cost variability by risk level and treatment

Building upon the risk-adjusted cost estimate model established in a prior study,^21^ patients were categorised into low, medium, and high-risk levels based on age and NIHSS scores upon hospital admission. Refer to the Data Analysis section for the specific methodology.

High-risk patients incurred an average total cost per patient of I$ 17,438 (SD I$ 19,781), whereas medium-risk patients averaged I$ 9926 (SD I$ 12,739), and low-risk patients averaged I$ 9572 (SD I$ 9673). The high-risk level was associated with significantly higher costs than low or medium-risk categories (p < 0.0001). Across all participating countries, mean costs exhibited an upward trend corresponding to patients' clinical risk levels. Treating high-risk patients incurred a substantial cost increase of I$ 7512 compared to medium-risk patients (p < 0.0001) and I$ 7866 compared to low-risk patients (p < 0.0001).

Additionally, this analysis revealed significant disparities in treatment costs among patients across different countries. The Brazilian centre demonstrated the highest mean medical treatment and IVT costs, with expenses reaching I$ 22,978 and I$ 28,688, respectively. With the highest potential for variability, the MT procedure showed the highest cost in Brazil, reaching I$ 60,701, and lowest cost in Costa Rica, at I$ 14,511. The centre in Argentina stood out with the highest cost for the combined treatment (54,906), while the centre in Colombia had the lowest cost (I$ 14,511), as detailed in Table 3. Additionally, specific observations were made regarding treatment access: the centre in Brazil and Mexico incurred notable expenses in MT. In contrast, the centre in Argentina registered higher costs associated with combined treatments. Moreover, limitations in treatment access were identified in certain centres, with Peru lacking access to MT and Chile's essential stroke centre offering solely IVT, with MT cases referred elsewhere.

**Table 3:** Differences in costs based on risk stratification levels per treatment.

Country	Treatments	Risk levels	N	Mean hospital length of stay days (SD)	Mean cost
All countries	Medical treatment	High risk	213 (19%)	16 (18)	I$ 13,552
		Medium risk	333 (30%)	8 (10)	I$ 8456
		Low risk	273 (25%)	8 (11)	I$ 8342
	IVT	High risk	85 (8%)	12 (12)	I$ 17,323
		Medium risk	65 (6%)	8 (7)	I$ 13,477
		Low risk	53 (5%)	8 (8)	I$ 12,984
	MT	High risk	30 (3%)	14 (21)	I$ 34,870
		Medium risk	15 (1%)	10 (7)	I$ 18,621
		Low risk	4 (0.4%)	9 (4)	I$ 23,795
	IVT + MT	High risk	23 (2%)	11 (11)	I$ 31,117
		Medium risk	9 (1%)	9 (2)	I$ 30,070
		Low risk	3 (0.3%)	8 (3)	I$ 23,310
					**I$ 12,203**
Argentina	Medical treatment	High risk	5 (3%)	7 (2)	I$ 5839
		Medium risk	73 (49%)	4 (4)	I$ 4642
		Low risk	43 (29%)	4 (3)	I$ 5655
	IVT	Medium risk	8 (5%)	4 (1)	I$ 9998
		Low risk	5 (3%)	4 (2)	I$ 11,957
	MT	High risk	5 (3%)	6 (3)	I$ 20,643
		Medium risk	3 (2%)	8 (2)	I$ 25,939
	IVT + MT	High risk	4 (3%)	12 (4)	I$ 49,617
		Medium risk	1 (1%)	57 (0)	I$ 91,723
		Low risk	1 (1%)	11 (0)	I$ 39,246
					**I$ 8487**
Brazil	Medical treatment	High risk	17 (11%)	14 (6)	I$ 31,999
		Medium risk	45 (29%)	11 (8)	I$ 22,707
		Low risk	37 (24%)	9 (5)	I$ 19,164
	IVT	High risk	16 (10%)	15 (9)	I$ 38,751
		Medium risk	16 (10%)	9 (4)	I$ 25,462
		Low risk	13 (8%)	9 (5)	I$ 20,275
	MT	High risk	5 (3%)	20 (8)	I$ 85,661
		Medium risk	5 (3%)	10 (5)	I$ 35,741
	IVT + MT	High risk	2 (1%)	17 (1)	I$ 62,315
		Medium risk	1 (1%)	38 (0)	I$ 18,145
					**I$ 27,488**
Chile	Medical treatment	High risk	24 (19%)	26 (23)	I$ 28,374
		Medium risk	30 (24%)	9 (9)	I$ 13,016
		Low risk	46 (37%)	7 (4)	I$ 10,568
	IVT	High risk	5 (4%)	14 (13)	I$ 21,448
		Medium risk	5 (4%)	6 (3)	I$ 12,177
		Low risk	13 (10%)	12 (11)	I$ 17,858
	MT	High risk	1 (%)	41 (0)	I$ 54,265
		Medium risk	1 (%)	4 (0)	I$ 13,408
		Low risk	1 (%)	9 (0)	I$ 19,876
					**I$ 16,233**
Colombia	Medical treatment	High risk	34 (22%)	11 (19)	I$ 6690
		Medium risk	42 (27%)	6 (7)	I$ 5713
		Low risk	28 (18%)	7 (10)	I$ 6718
	IVT	High risk	10 (7%)	9 (9)	I$ 10,819
		Medium risk	7 (5%)	6 (5)	I$ 9063
		Low risk	6 (4%)	3 (2)	I$ 6634
	MT	High risk	10 (7%)	8 (5)	I$ 18,030
		Medium risk	3 (2%)	5 (3)	I$ 14,209
		Low risk	3 (2%)	9 (4)	I$ 18,203
	IVT + MT	High risk	7 (5%)	6 (4)	I$ 6924
		Medium risk	2 (%)	6 (2)	I$ 3976
		Low risk	1 (%)	8 (0)	I$ 4478
					**I$ 8673**
Costa Rica	Medical treatment	High risk	59 (30%)	12 (13)	I$ 6924
		Medium risk	53 (27%)	4 (3)	I$ 3976
		Low risk	36 (18%)	7 (10)	I$ 4478
	IVT	High risk	18 (9%)	5 (4)	I$ 5539
		Medium risk	10 (5%)	3 (1)	I$ 4768
		Low risk	5 (3%)	2 (0)	I$ 6225
	MT	High risk	4 (2%)	5 (1)	I$ 15,743
		Medium risk	1 (1%)	1 (0)	I$ 9583
	IVT + MT	High risk	7 (4%)	12 (16)	I$ 23,659
		Medium risk	4 (2%)	6 (3)	I$ 12,677
					**I$ 6334**
Mexico	Medical treatment	High risk	26 (25%)	13 (15)	I$ 19,307
		Medium risk	20 (19%)	8 (26)	I$ 6646
		Low risk	25 (24%)	2 (3)	I$ 4748
	IVT	High risk	18 (17%)	10 (8)	I$ 18,113
		Medium risk	7 (7%)	4 (3)	I$ 10,215
		Low risk	3 (3%)	2 (1)	I$ 7187
	MT	High risk	3 (3%)	41 (49)	I$ 56,204
	IVT + MT	Low risk	1 (1%)	7 (0)	I$ 31,263
					**I$ 13,326**
Peru	Medical treatment	High risk	37 (31%)	19 (15)	I$ 7121
		Medium risk	45 (38%)	12 (4)	I$ 4982
		Low risk	26 (22%)	10 (5)	I$ 4824
	IVT	High risk	3 (3%)	23 (11)	I$ 11,454
		Medium risk	6 (5%)	11 (4)	I$ 7213
		Low risk	2 (2%)	10 (1)	I$ 5806
					**I$ 5902**
Uruguay	Medical treatment	High risk	11 (11%)	31 (29)	I$ 20,989
		Medium risk	24 (23%)	19 (15)	I$ 8932
		Low risk	32 (31%)	19 (24)	I$ 8644
	IVT	High risk	15 (15%)	22 (19)	I$ 11,794
		Medium risk	7 (7%)	20 (12)	I$ 11,398
		Low risk	6 (6%)	15 (6)	I$ 9114
	MT	High risk	2 (2%)	15 (13)	I$ 24,210
		Medium risk	2 (2%)	24 (4)	I$ 17,394
	IVT + MT	High risk	3 (3%)	18 (11)	I$ 31,965
		Medium risk	1 (1%)	15 (0)	I$ 17,459
					**I$ 11,940**

The numbers in bold are the mean of each category. Above, groups without patient entries are excluded from the table. Low-risk level: patients under 70 years with an NIHSS score <8; Medium-risk level: patients under 70 years with an NIHSS score between 8 and 15, as well as those older than 70 with an NIHSS score <8; High-risk level: patients older than 70 years with an NIHSS score >8 and those with an NIHSS score >15.

IVT = Intravenous Trombolysis; MT = Mechanical Thrombectomy; mRS = modified Rankin Score; SD = Standard Deviation; NA = Not applicable.

### Cost drivers and variabilities in stroke centres and treatment modalities

The length of hospital stay is the primary cost driver in stroke centres, directly impacting medication consumption, diagnostic tests, hospital infrastructure usage, and visits from staff. The Argentinian centre achieved the lowest mean times among the observed variabilities across all treatments and risk levels. In contrast, the Uruguayan centre demonstrates longer hospital stays for medical treatment and IVT, while the Brazilian centre notably exhibits prolonged hospitalisation for combined treatment.

The hospital structure emerged as one of the most expensive cost components across countries and treatments, influenced by the in-hospital processes adopted by each centre (Fig. 1). The stroke centres from Argentina, Costa Rica, Uruguay, and Colombia exhibit a similar pattern, characterised by shorter lengths of stay in the emergency department and the proportion of time spent in the ward and stroke units. A different behaviour is encountered in stroke centres in Brazil and Chile, where emergency departments and stroke units concentrate on longer periods of hospitalisation. In addition to the care pathways variabilities observed, structural costs attributed to each unit also contributed to the cost differences measured. For instance, the stroke centre in Chile incurred higher depreciation, energy, and support expenses, while Peru had the lowest depreciation and support supply costs.

Labour costs were a significant cost driver, showing important variabilities across all countries. The centre in Chile has the highest labour cost rate, followed by the centre in Brazil, with the centre in Colombia exhibiting high rates, particularly for specialised professionals. The lowest unit cost rates were observed in the centre in Uruguay for specialised professionals and in the centre in Colombia for non-specialized ones. Among the exams, brain computed tomography scans showed a disparity of up to five times between the lowest cost (Chile, I$ 46) and the highest (Uruguay, I$ 225). The variability in IVT treatment costs follows a similar pattern, with the centre of Costa Rica having the lowest cost (I$ 400) and the centre of Argentina the highest (I$ 1872). Tenecteplase (I$ 1767) was administered to 14 out of 203 IVT patients. Supplementary Material V contains the cost composition information per country, risk level, and therapy for detailed cost information. A table summarizing the costs in medians and interquartile ranges is provided in Supplementary Material VI.

## Discussion

This study aimed to evaluate the direct costs of acute ischemic stroke treatment in Latin America using TDABC, highlighting cost variability across countries and treatment modalities. Significant differences were observed, with direct costs ranging from I$ 5902 in Peru to I$ 27,488 in Brazil, underscoring the economic disparities in stroke care across the region. The mean costs for different treatments were as follows: combined IVT plus MT at I$ 29,016, MT alone at I$ 30,153, medical treatment at I$ 9,735, and IVT at I$ 14,930. These findings provide a detailed view of the economic impact of stroke management in Latin America, offering essential insights for policymakers and healthcare providers to enhance care delivery and reduce healthcare system burdens.^23^^,^^24^

Cost variations in acute ischemic stroke care across Latin America can be attributed to differences in care protocols, healthcare systems, access to treatments, professional capacity, varying lengths of hospital stays, stroke severity, and costs of exams, medications, and treatments.^25^^,^^26^ For example, Chile and Brazil demonstrated the highest medical treatment costs, partly due to higher depreciation, energy, and support expenses, and longer periods of hospitalization in emergency departments and stroke units, while Costa Rica had the lowest costs for MT procedures. Argentina had the highest cost for combined treatment, and Colombia had the lowest. Additionally, longer hospital stays, a primary cost driver, were observed in Uruguay, whereas Argentina had shorter stays. Reflecting the unique circumstances of each country's healthcare system, these variations underscore the imperative for targeted interventions to address disparities and enhance healthcare delivery. As treatment complexity rises, fewer patients can receive reperfusion therapies, highlighting the importance of raising public awareness about stroke and emphasizing the need for enhanced coordination between pre-hospital and hospital systems in stroke care.^11^ Extending the availability of 10 and 20 mg of rtPA in Latin American countries, mirroring the practice in Brazil, can reduce treatment costs minimizing medication wastage.^27^ Furthermore, despite their higher costs, advanced treatments like MT and combined IVT plus MT have been associated with improved outcomes, as evidenced by higher rates of improvement in NIHSS and mRS scores among recipients.^9^

Stratifying costs according to risk levels (high, medium, and low) reveals significant variation per patient among countries, offering insights into the correlation between clinical profiles, treatment received, and projected treatment costs. A global concern emerges from the increasing stroke incidence among younger patients, particularly in LMICs.^3^^,^^4^ The predominance of moderate-risk patients, largely due to their younger age rather than NIHSS scores, underscores the urgent need for investments in awareness and prevention efforts to mitigate future stroke burdens over the next 30 years.^5^ Global collaboration is essential to ensure widespread access to life-saving stroke treatments. Recently, initiatives led by the World Stroke Organization and the Iberoamerican Stroke Society have focused on enhancing stroke care implementation in Latin America. This has been achieved through the collaborative efforts of the Global Stroke Alliance Conferences and the Latin American Stroke Ministerial Meeting,^4^ reflecting a strong commitment to improving accessibility to stroke prevention, treatment, and rehabilitation. Addressing these disparities through these initiatives is paramount to ensuring equitable access to essential stroke interventions and alleviating the financial strain on healthcare systems in LMICs.^28^

Implementing risk and outcomes-adjusted mechanisms for reimbursing healthcare systems to optimise public spending necessitates a thorough understanding of the factors influencing cost variations.^28^ Proposed pilot studies exploring innovative provider payment systems present promising avenues to tackle this challenge.^29^ Understanding the detailed costs of stroke care has significant implications for the countries included in our study. The findings can inform policy initiatives to optimize resource allocation and improve efficiency for countries with comprehensive public health systems like Chile, Uruguay, and Brazil. For example, Chile can address higher costs from longer hospital stays and structural expenses, while Uruguay can explore reducing unnecessary hospital days. Even in countries with fragmented systems, like Mexico, detailed cost information helps identify high-cost areas and standardise care protocols to improve cost management. Introducing changes to hospital policies, focused on standardising resource utilisation and minimising variation in length of stay, could effectively curb costs and enhance efficiencies in acute stroke management. The efficacy of these strategies is likely to be bolstered by broader policy initiatives currently underway to reform hospital reimbursement systems.^29^ Collaborative initiatives led by local health authorities should prioritise evidence-based reperfusion therapies within preventive strategies, integrating economic considerations to address evolving stroke challenges, enhance patient outcomes, and mitigate societal and economic burdens. This approach aids decision-making processes by informing resource allocation strategies and underscores the importance of integrating value principles into healthcare financing strategies.

Overall, the findings of this study point to the need for targeted interventions to address disparities in stroke care access and outcomes across Latin America. By leveraging standardised costing methods and VBHC strategies, policymakers and healthcare providers can work collaboratively to optimise resource allocation and improve stroke care delivery. However, further research is warranted to explore the long-term clinical and economic implications of stroke management in the region, considering both direct and indirect costs. Additionally, efforts to enhance access to life-saving interventions like MT must be prioritised to ensure equitable healthcare delivery for all stroke patients, regardless of geographical location or socioeconomic status.

Our study has limitations, including the limited representativeness of the distinct stroke centre (comprehensive vs primary) and hospital (private, public, academic) types. Moreover, it focuses solely on direct and acute hospitalisation costs. Additionally, our study's selection of stroke centres based on convenience may have resulted in a biased sample of hospitals, potentially not fully representing the diversity of stroke care costs in Latin America due to variations in labour costs and infrastructure among different regions. Most of the countries included in this study are classified as having relatively higher economic resources within the region, which may limit the generalisability of our findings to less affluent settings. The inclusion of only one centre per country further restricts the generalisability of the results and prevents an analysis of cost variability across different facility types (public, private, and academic) within individual countries Our study assumed a uniform care flow across all centres. While this facilitated our analysis, it may not fully account for variations in stroke care delivery between different countries and centres. Despite these limitations, the study highlights standardised micro-costing techniques, which are recognised as the gold standard in health economic analyses. The cost results from 8 Latin American countries suggest that the standardised framework employed here could be replicated worldwide, enhancing cost information granularity and facilitating more effective resource distribution policies in LMICs where evidence remains limited. Future research should investigate the variabilities in cost acquisition for specific items like medications and materials and explore socio-determinants of health such as sex, socioeconomic status, race, ethnicity, and age to identify variations in costs and outcomes across the region. Additionally, future studies should examine the implications of indirect costs, long-term follow-up costs, and clinical outcomes associated with stroke management in the region.

### Conclusion

This is Latin America's largest real-world data-based focusing on the costs of acute ischemic stroke treatment in Latin America. Our findings provide crucial guidance for healthcare policy and resource allocation, particularly contributing to expanding access to reperfusion treatments. By elucidating the factors driving cost variability and their implications, our findings contribute to the ongoing efforts to enhance the accessibility, affordability, and quality of stroke care in the region. Continued investment in evidence-based research and policy interventions from all stakeholders is key to ensuring equitable access to optimal stroke care and reducing stroke-related disabilities and deaths in Latin America.

## Contributors

ACS, APE, GS, CP, LBD, ER, PA, MB and SM conceptualized the structure and design of the manuscript. LBD, APE, MM conducted the main data analysis. LBD wrote the first draft of the manuscript, and ACS, APE, CP, SM, GN, and RN revised all sections and wrote the final version. All other coauthors provided critical intellectual contributions, contributed to the overall structure and concepts, and approved the final version.

ACS, APBSE, and JSS verified the data. ACS and LBD had access to raw data, and APBSE and ACS had the final responsibility for the decision to submit it for publication.

## Data sharing statement

The data supporting this study's findings are available from the corresponding author upon reasonable request. All data shared will be anonymized to protect participant privacy.

## Declaration of generative AI and AI-assisted technologies in the writing process

During the preparation of this work the authors used ChatGPT (GPT-4 version) to review and ensure clarity and coherence of the manuscript. After using this tool, the authors reviewed and edited the content as needed and take full responsibility for the content of the publication.

## Declaration of interests

ACS has received speaker fees from BI and limited grants from the World Stroke Organization and BI. PA has received speaker fees from BI, Ipsen, and Abbott. LAC reports receiving limited grants from the World Stroke Organization and BI/Angels Initiative and consulting and speaker fees from Allm, AstraZeneca, Boehringer Ingelheim, and ISchemaView, outside of this work. RGN reports consulting fees for advisory roles with Anaconda, Biogen, Cerenovus, Genentech, Philips, Hybernia, Hyperfine, Imperative Care, Medtronic, Phenox, Philips, Prolong Pharmaceuticals, Stryker Neurovascular, Shanghai Wallaby, Synchron, and stock options for advisory roles with Astrocyte, Brainomix, Cerebrotech, Ceretrieve, Corindus Vascular Robotics, CrestecBio Inc., Euphrates Vascular, Inc., Vesalio, Viz-AI, RapidPulse and Perfuze. RGN is one of the Principal Investigators of the “Endovascular Therapy for Low NIHSS Ischemic Strokes (ENDOLOW)” trial. Funding for this project is provided by Cerenovus. RGN is the Principal Investigator of the “Combined Thrombectomy for Distal MediUm Vessel Occlusion StroKe (DUSK)” trial. Funding for this project is provided by Stryker Neurovascular. RGN is an investor in Viz-AI, Perfuze, Cerebrotech, Reist/Q’Apel Medical, Truvic, Tulavi Therapeutics, Vastrax, Piraeus Medical, Brain4Care, Quantanosis AI, and Viseon. GS received a modest compensation from the World Stroke Organization for his role of Executive Director/Editor-in-Chief of the World Stroke Academy (WSA). SM reports funding from the Brazilian Ministry of Health for the Resilient and PROMOTE trials and speaker fees from Boehringer Ingelheim, Pfizer, Bayer, Medtronic, Penumbra, Novartis, Novo Nordisk, Servier, Daiichi Sankyo, and Astra Zeneca.

## Appendix ASupplementary data

Supplementary Material IV

Supplementary Materials I–III, V, and VI

Translated Summary_ESP

Translated Summary_POR
