# Supplementary material for: Cost evaluation of acute ischemic stroke in Latin America: a multicentric study
Source: Lancet Reg Health Am. 2024 Dec 6;41:100959. doi: 10.1016/j.lana.2024.100959 (PMC11665535; doi:10.1016/j.lana.2024.100959)
Supplement: Supplementary Materials I–III, V, and VI [file mmc2.pdf]

Supplementary Material Files

Supplementary Material I: Research Conduction
Supplementary Material II: The steps to apply the TDABC method
Supplementary Material III: Departmental Length of Stay
Supplementary Material IV: Separate file
Supplementary Material V: Cost Composition Information Per Country, Risk Level, and Therapy
Supplementary Material VI: Median and Interquartile Range Values of Costs Based on Treatments by Center and Costs Based on Treatment by Risk Levels

Supplementary Material I: Research Conduction

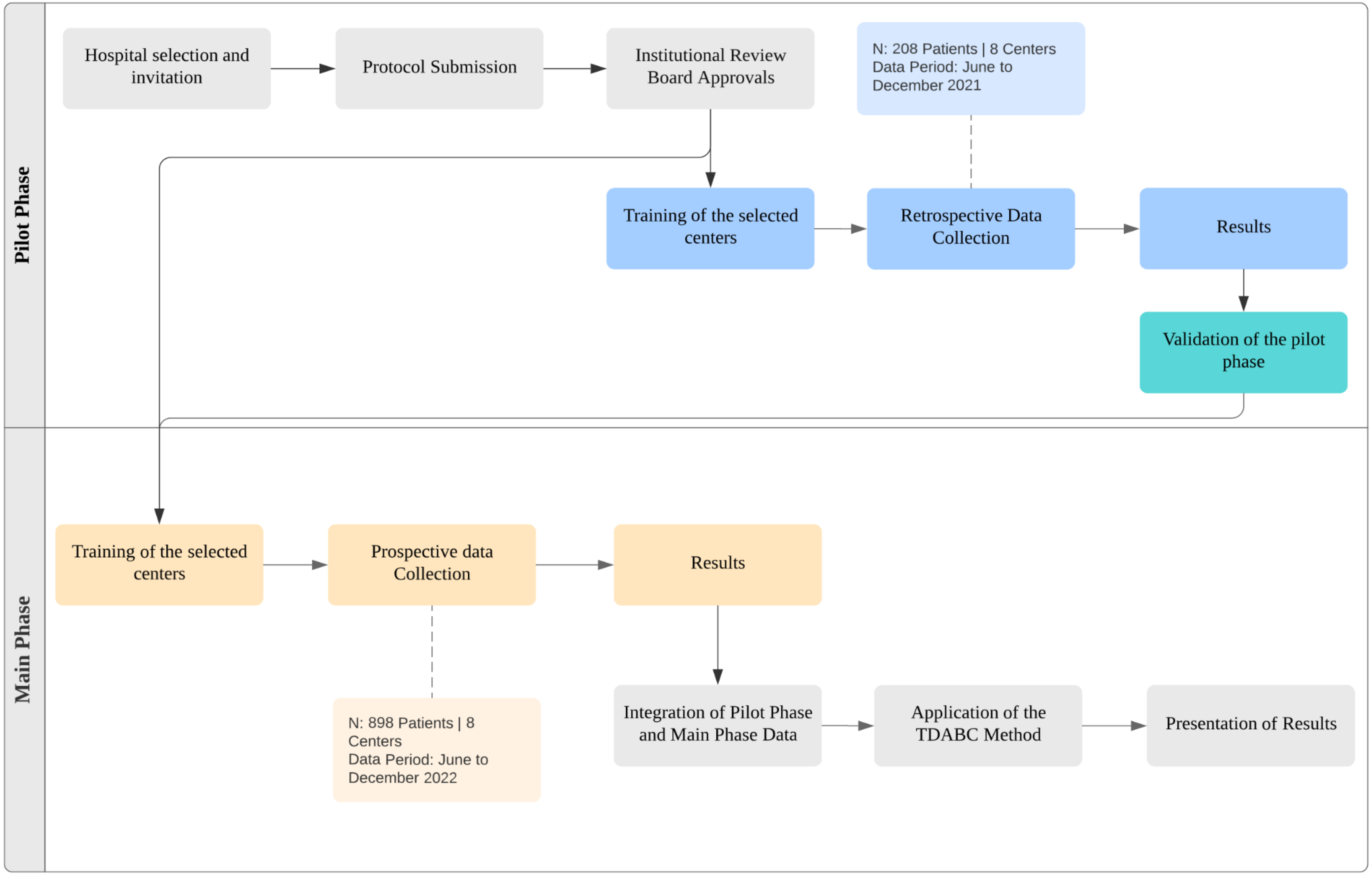

Supplementary Material II: The steps to apply the TDABC method

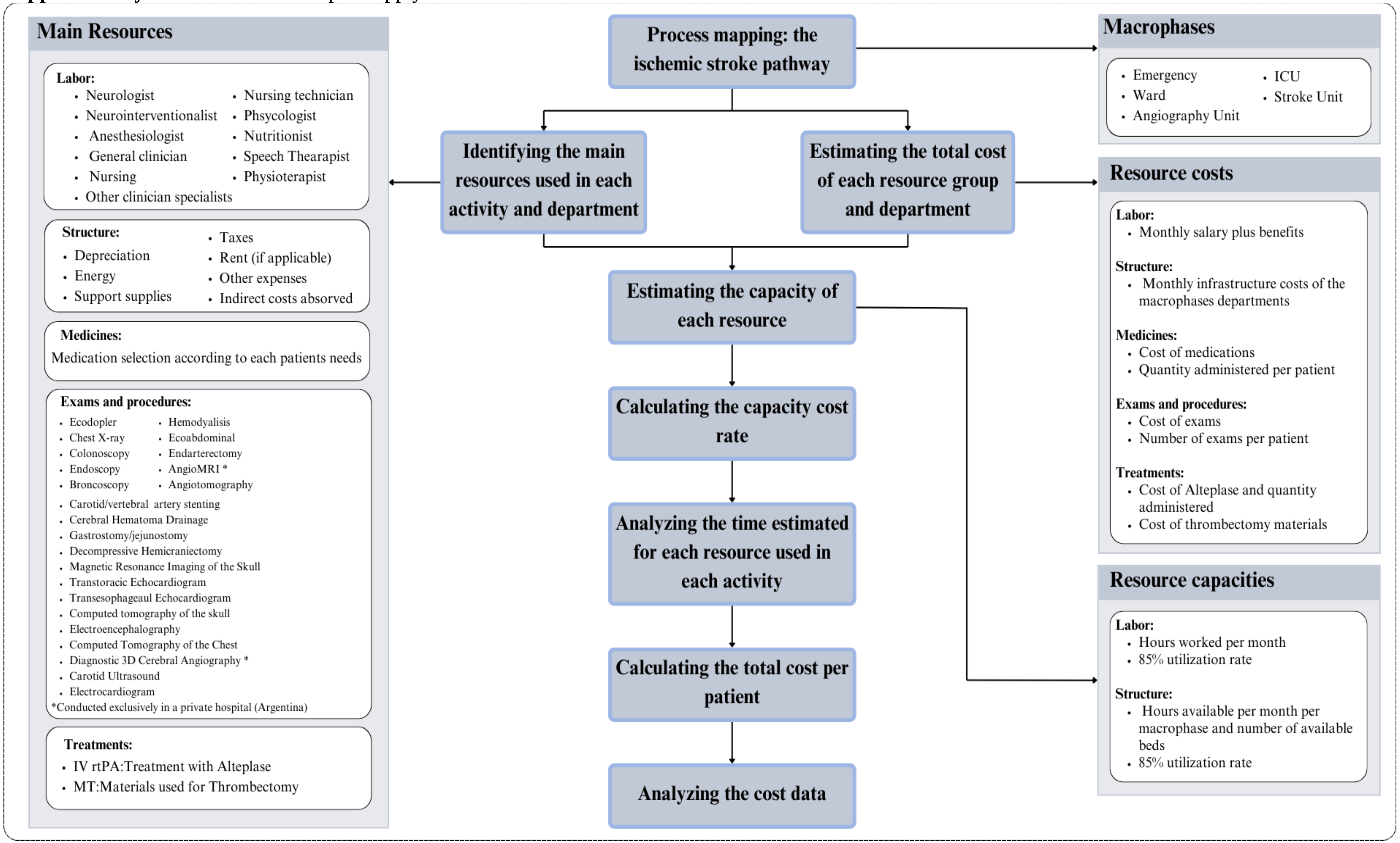

Supplementary Material III: Departmental Length of Stay

	Mean LOT Emergency (SD)	Mean LOT Ward (SD)	Mean LOT Angiography Unit (SD)	Mean LOT ICU (SD)	Mean LOT Stroke Unit (SD)
Argentina	4.6 (4.7)	98.9 (322.1)	2.7 (0.8)	86.5 (47.5)	61 (45.4)
Brazil	73.2 (67.1)	103.2 (84.2)	2 (1.1)	89.5 (64)	170 (105.8)
Chile	61.2 (34.5)	0.5 (-)	1 (-)	48.5 (67,2)	232.6 (303.8)
Colombia	7.0 (18.4)	121.6 (189.4)	2.6 (3.6)	82.4 (67.1)	110.7 (119.6)
Costa Rica	2.4 (3.1)	347.2 (298.4)	1.4 (0.5)	49.9 (46.4)	82.8 (35.7)
Mexico	76.1 (75.3)	343.9 (416)	2.6 (0.9)	236.6 (185.6)	NA
Peru	14 (13.4)	154.2 (88.6)	1.7 (0.6)	389.1 (272)	259.2 (152)
Uruguay	21.2 (17.8)	393.3 (374.8)	2.2 (0.9)	298.1 (499)	138.9 (103.8)

Legend: SD: Standard Deviation; NA = Not applicable; LOT = Length of Time
All values in the table are in hours.

Supplementary Material IV: Separate file

Supplementary Material V: Cost Composition Information Per Country, Risk Level, and Therapy.

								**Structure Costs**					
Country	**Treatments***	**Risk Levels**	**Labor Costs**	**Thrombolysis**	**Materials thrombectomy**	**Medicines**	**Exams and procedures**	**Emergency**	**Ward**	**Angiography**	**ICU**	**Stroke unit**	**Total Cost**
Arg	1	High	2352 (999)	-	-	509 (694)	1353 (211)	39 (16)	544 (439)	-	-	1044 (560)	5839
Arg	1	Medium	1683 (1481)	-	-	448 (2547)	1279 (506)	42 (33)	319 (484)	-	19 (167)	823 (602)	4642
Arg	1	Low	2030 (2260)	-	-	179 (274)	1550 (901)	48 (56)	927 (4272)	-	-	920 (615)	5655
Arg	2	Medium	1891 (553)	4419 (2336)	-	417 (586)	1419 (443)	51 (74)	362 (365)	25 (66)	-	1048 (684)	9998
Arg	2	Low	2018 (706)	5572 (1668)	-	490 (663)	1403 (482)	37 (14)	757 (591)	-	483 (1079)	1197 (849)	11957
Arg	3	High	8194 (4378)	-	6872 (3844)	887 (714)	2067 (1079)	23 (10)	554 (923)	165 (165)	206 (460)	1676 (1645)	20643
Arg	3	Medium	10434 (1521)	-	8757 (807)	670 (350)	2208 (194)	62 (69)	1450 (422)	155 (268)	329 (569)	1874 (1618)	25939
Arg	4	High	12855 (2522)	6029 (1523)	9683 (7157)	13541 (17331)	2496 (953)	19 (20)	982 (787)	492 (164)	1632 (1698)	1888 (2538)	49617
Arg	4	Medium	33270 (-)	6790 (-)	15674 (-)	13519 (-)	3631 (-)	14 (-)	16748 (-)	544 (-)	1452 (-)	94 (-)	91723
Arg	4	Low	11887 (-)	6790 (-)	9283 (-)	2644 (-)	2642 (-)	3 (-)	698 (-)	544 (-)	2949 (-)	1796 (-)	39246
													**8487**
Br	1	High	14982 (10006)	-	-	323 (352)	432 (268)	2432 (1566)	8 (33)	20 (81)	-	13802 (4975)	31999
Br	1	Medium	10712 (15603)	-	-	294 (454)	389 (221)	2319 (2810)	398 (1128)	2 (16)	334 (1374)	8259 (6748)	22707
Br	1	Low	7663 (8243)	-	-	242 (353)	503 (287)	2190 (1218)	130 (442)	25 (67)	346 (860)	8065 (5665)	19164
Br	2	High	18562 (21224)	1472 (278)	-	1399 (619)	529 (297)	2411 (1216)	82 (327)	-	1278 (3510)	13020 (7436)	38751
Br	2	Medium	9704 (12594)	1578 (222)	-	2375 (5265)	380 (203)	1543 (1207)	123 (493)	16 (46)	783 (1632)	8960 (6239)	25462
Br	2	Low	7143 (9717)	1536 (248)	-	1055 (816)	574 (275)	2016 (868)	177 (432)	-	-	7773 (5925)	20275
Br	3	High	49385 (38322)	-	7066 (0)	391 (332)	465 (240)	194 (118)	-	229 (143)	7611 (5274)	20319 (7397)	85661
Br	3	Medium	13278 (10058)	-	7066 (0)	376 (322)	447 (262)	219 (140)	-	249 (50)	4681 (2278)	9425 (5146)	35741
Br	4	High	28081 (2989)	1385 (194)	7066 (0)	1835 (129)	740 (7)	359 (34)	-	367 (74)	4993 (2287)	17489 (1760)	62315
Br	4	Medium	2505 (-)	1522 (-)	7066 (-)	107 (-)	134 (-)	1512 (-)	295 (-)	210 (-)	-	4794 (-)	18145

													**27488**
Chi	1	High	17763 (11731)	-	-	80 (28)	688 (304)	1571 (705)	0 (2)	23 (114)	-	8250 (7987)	28374
Chi	1	Medium	8598 (6553)	-	-	73 (16)	537 (288)	1796 (889)	-	-	-	2012 (2511)	13016
Chi	1	Low	6752 (3002)	-	-	76 (24)	428 (165)	1466 (909)	-	-	1 (4)	1845 (1365)	10568
Chi	2	High	12982 (10009)	265 (0)	-	72 (6)	847 (362)	1943 (1378)	-	-	-	3997 (5082)	21448
Chi	2	Medium	6081 (2292)	289 (0)	-	68 (4)	508 (119)	2062 (1311)	-	-	-	1849 (1321)	12177
Chi	2	Low	10593 (8908)	319 (0)	-	75 (24)	774 (431)	1613 (891)	-	-	199 (716)	3072 (3965)	17858
Chi	3	High	29674 (-)	-	7066 (-)	66 (-)	1589 (-)	196 (-)	-	-	-	15674 (-)	54265
Chi	3	Medium	4015 (-)	-	7066 (-)	65 (-)	327 (-)	759 (-)	-	-	-	1176 (-)	13408
Chi	3	Low	7895 (-)	-	7066 (-)	76 (-)	448 (-)	2050 (-)	-	-	-	2351 (-)	19876
													**16233**
Col	1	High	1060 (662)	-	-	478 (784)	1295 (572)	110 (282)	135 (788)	99 (323)	2288 (1674)	1225 (1231)	6690
Col	1	Medium	872 (594)	-	-	340 (738)	1421 (745)	155 (341)	311 (740)	213 (490)	1611 (1298)	790 (1544)	5713
Col	1	Low	911 (833)	-	-	336 (596)	1417 (434)	137 (272)	536 (2324)	653 (2501)	1697 (1912)	1030 (1779)	6718
Col	2	High	1452 (1178)	1849 (436)	-	888 (520)	1555 (575)	376 (1014)	35 (112)	112 (236)	2934 (2575)	1618 (2357)	10819
Col	2	Medium	953 (270)	1911 (393)	-	1420 (1130)	1271 (525)	48 (33)	357 (795)	160 (274)	2346 (1863)	595 (815)	9063
Col	2	Low	551 (355)	1532 (560)	-	1098 (392)	1449 (494)	45 (20)	-	-	1577 (1543)	383 (348)	6634
Col	3	High	1842 (828)	-	8107 (2619)	869 (750)	1576 (408)	35 (25)	11 (34)	1330 (1075)	3379 (2124)	881 (1366)	18030
Col	3	Medium	2110 (996)	-	3684 (1395)	1051 (1147)	1843 (250)	189 (261)	326 (565)	923 (1148)	3656 (4757)	427 (489)	14209
Col	3	Low	1435 (516)	-	8919 (1186)	907 (745)	1737 (326)	147 (173)	349 (604)	555 (552)	3621 (2655)	534 (667)	18203
Col	4	High	1663 (646)	1904 (390)	8266 (2021)	1188 (576)	1338 (343)	39 (26)	43 (113)	1157 (1186)	2120 (965)	1007 (1313)	18725
Col	4	Medium	1330 (210)	2043 (0)	5261 (3506)	1426 (290)	1883 (655)	48 (13)	-	828 (1171)	1936 (0)	1121 (680)	15877
Col	4	Low	2280 (-)	2043 (-)	5353 (-)	1842 (-)	2059 (-)	57 (-)	-	2208 (-)	1936 (-)	1922 (-)	19700
													**16233**
Cos	1	High	1712 (1511)	-	-	NA	1358 (739)	58 (87)	3328 (4008)	-	94 (723)	375 (589)	6924
Cos	1	Medium	992 (876)	-	-	NA	1131 (1555)	50 (45)	1072 (2472)	-	-	730 (618)	3976
Cos	1	Low	1083 (876)	-	-	NA	1396 (1912)	63 (43)	1018 (2433)	-	-	919 (596)	4478

Cos	2	High	1177 (751)	800 (0)	-	NA	1478 (725)	8 (6)	810 (1698)	71 (207)	72 (304)	1124 (587)	5539
Cos	2	Medium	795 (206)	800 (0)	-	NA	2061 (3603)	10 (7)	146 (461)	-	-	957 (467)	4768
Cos	2	Low	740 (188)	800 (0)	-	NA	3618 (5087)	31 (52)	-	-	-	1035 (231)	6225
Cos	3	High	1551 (856)	-	6915 (2355)	NA	4102 (5931)	9 (4)	364 (729)	598 (465)	806 (812)	1398 (952)	15743
Cos	3	Medium	1008 (-)	-	6051 (-)	NA	556 (-)	19 (-)	-	662 (-)	645 (-)	641 (-)	9583
Cos	4	High	2044 (1300)	800 (0)	8479 (1480)	NA	8113 (6126)	12 (10)	1118 (2053)	862 (349)	553 (784)	1678 (1191)	23659
Cos	4	Medium	1082 (318)	800 (0)	6701 (2091)	NA	1239 (274)	6 (3)	729 (1457)	593 (83)	806 (323)	721 (842)	12677
													**6334**
Mex	1	High	12126 (9721)	-	-	1043 (3125)	675 (346)	2021 (1724)	2827 (4403)	-	614 (1788)	-	19307
Mex	1	Medium	4355 (5387)	-	-	25 (46)	435 (238)	881 (807)	789 (3187)	-	161 (721)	-	6646
Mex	1	Low	3191 (4076)	-	-	76 (239)	425 (186)	784 (1062)	227 (638)	45 (224)	-	-	4748
Mex	2	High	10583 (7371)	2161 (39)	-	673 (1324)	568 (164)	2272 (1621)	1354 (1742)	-	501 (1463)	-	18113
Mex	2	Medium	5753 (3384)	2123 (0)	-	21 (31)	586 (164)	1440 (671)	291 (771)	-	-	-	10215
Mex	2	Low	3683 (1665)	2148 (44)	-	11 (14)	419 (97)	927 (443)	-	-	-	-	7187
Mex	3	High	22829 (23086)	-	14991 (304)	184 (154)	836 (633)	2318 (2828)	7577 (13124)	1662 (543)	5807 (10057)	-	56204
Mex	4	Low	9814 (-)	2123 (-)	14815 (-)	10 (-)	523 (-)	2290 (-)	583 (-)	1104 (-)	-	-	31263
													**13326**
Per	1	High	1670 (1047)	-	-	193 (330)	1057 (239)	257 (247)	29 (42)	-	177 (789)	3739 (2516)	7121
Per	1	Medium	1153 (422)	-	-	73 (147)	1061 (245)	144 (107)	24 (39)	64 (428)	54 (365)	2408 (1381)	4982
Per	1	Low	964 (379)	-	-	24 (38)	1088 (245)	136 (116)	10 (23)	166 (619)	-	2437 (1403)	4824
Per	2	High	2233 (1279)	2216 (35)	-	180 (123)	1165 (134)	268 (324)	27 (46)	-	3028 (5244)	2338 (900)	11454
Per	2	Medium	1154 (374)	2216 (35)	-	118 (186)	1218 (199)	286 (152)	18 (23)	-	-	2202 (722)	7213
Per	2	Low	795 (155)	1677 (791)	-	5 (0)	1011 (117)	128 (124)	-	-	-	2191 (273)	5806
													**5902**
Uru	1	High	2417 (3668)	-	-	403 (446)	1726 (1584)	680 (644)	3782 (4388)	-	10518 (19549)	1463 (2585)	20989
Uru	1	Medium	1098 (918)	-	-	179 (255)	1341 (1234)	366 (364)	4596 (4603)	-	368 (1358)	983 (1055)	8932
Uru	1	Low	1469 (1729)	-	-	63 (100)	1499 (1725)	352 (247)	3850 (5666)	-	437 (1461)	975 (917)	8644

Uru	2	High	1920 (1668)	1410 (19)	-	133 (105)	1417 (390)	282 (239)	4583 (4690)	-	523 (957)	1526 (1833)	11794
Uru	2	Medium	2126 (1182)	1402 (27)	-	77 (33)	1417 (320)	458 (261)	3708 (3426)	-	-	2210 (1620)	11398
Uru	2	Low	1054 (261)	1390 (30)	-	36 (25)	1341 (350)	296 (251)	3488 (1811)	-	-	1510 (705)	9114
Uru	3	High	2143 (2610)	-	9097 (3876)	304 (388)	1856 (1426)	29 (13)	-	1104 (781)	9678 (11861)	-	24210
Uru	3	Medium	2595 (311)	-	4960 (-)	79 (68)	1381 (30)	-	5828 (2061)	1104 (0)	645 (0)	801 (680)	17394
Uru	4	High	1363 (929)	7645 (5928)	10900 (2472)	299 (351)	1624 (687)	50 (26)	1304 (2259)	1393 (737)	5044 (5177)	2342 (4057)	31965
Uru	4	Medium	2383 (-)	1417 (-)	4752 (-)	51 (-)	2651 (-)	-	291 (-)	1104 (-)	645 (-)	4164 (-)	17459
												**11940**

Legend: IVT = Intravenous Trombolysis; MT = Mechanical Thrombectomy; mRS = modified Rankin Score; NA = do not apply, no medication costs. The groups not listed in the table are those in which there are no patients. 1= Medical Treatment; 2 = IVT; 3 = MT; 4 = IVT + MT.

Supplementary Material VI: Median and Interquartile Range Values of Costs Based on Treatments by Center and Costs Based on Treatment by Risk Levels

Country	Treatments	Risk	Median	IQR 25	IQR 75
**All Countries**	Medical Treatment	High Risk	I$ 7,850	I$ 4,684	I$ 15,433
		Medium Risk	I$ 4,790	I$ 3,197	I$ 8,165
		Low Risk	I$ 5,235	I$ 3,446	I$ 10,159
	IVT	High Risk	I$ 11,336	I$ 6,158	I$ 20,795
		Medium Risk	I$ 9,893	I$ 6,617	I$ 13,478
		Low Risk	I$ 8,976	I$ 6,673	I$ 15,586
	MT	High Risk	I$ 20,717	I$ 15,370	I$ 35,458
		Medium Risk	I$ 22,957	I$ 14,789	I$ 26,791
		Low Risk	I$ 17,876	I$ 15,843	I$ 20,654
	IVT + MT	High Risk	I$ 27,669	I$ 18,166	I$ 32,539
		Medium Risk	I$ 15,571	I$ 14,142	I$ 17,612
		Low Risk	I$31,263	I$ 25,481	I$ 35, 255

Legend: IVT = Intravenous Trombolysis; MT = Mechanical Thrombectomy. NA = not applicable; IQR25;75: first and third quartiles.
