# Supplementary material for: Cost evaluation of acute ischemic stroke in Latin America: a multicentric study
Source: Lancet Reg Health Am. 2024 Dec 6;41:100959. doi: 10.1016/j.lana.2024.100959 (PMC11665535; doi:10.1016/j.lana.2024.100959)
Supplement: Translated Summary_ESP [file mmc3.pdf]

__Editorial disclaimer:__ This translation in Spanish was submitted by the authors and we reproduce it as supplied. It has not been peer-reviewed. Our editorial processes have only been applied to the original abstract in English, which should serve as a reference for this manuscript.

Resumen

Antecedentes: La literatura actual destaca una brecha en los datos precisos sobre los costos del accidente cerebrovascular (ACV) en América Latina. Este estudio mide los costos reales asociados con la atención del ACV isquémico agudo en América Latina utilizando Costeo Basado en Actividades y Tiempo (TDABC). Los hallazgos tienen como objetivo establecer una base sólida para la adopción de estrategias de atención médica basada en el valor (VBHC) en la región.

Métodos: Estudio de análisis observacional, multicéntrico e internacional de los costos directos y desenlaces en pacientes hospitalizados por ACV isquémico agudo desde diciembre de 2021 hasta diciembre de 2022. Se analizaron datos de centros de ACV en Argentina, Brasil, Chile, Colombia, Costa Rica, México, Perú y Uruguay. Los costos fueron estratificados por país. Se consideraron factores como desenlace favorable según la escala de Rankin modificada (mRS 0-2), niveles de riesgo clínico e intervenciones de tratamiento. Se utilizaron modelos de Ecuación de Estimación Generalizada(GEE) para evaluar la relación entre las variables clínicas y el costo total por paciente.

Resultados: Se incluyeron un total de 1,106 pacientes en el estudio. Entre ellos, el 74% recibió solamente tratamiento médico, el 18% recibió Trombolisis Intravenosa (IVT), el 4% se sometió a Trombectomía Mecánica (MT) y el 3% recibió terapia combinada (IVT+MT). El costo promedio por paciente fue de I$12,203 (SD I$15,055), con un 49% logrando un desenlace funcional favorable. En comparación con el tratamiento médico únicamente, la MT generó costos 3,1 veces mayores, con un costo incremental de I$ 20,418 por paciente ($p < 0,0001$). En todos los países, los costos aumentaron según la severidad clínica delos pacientes y los tipos de tratamiento, siendo la duración de la estancia hospitalaria el principal factor de aumento del costo.

Interpretación: Nuestro estudio destaca disparidades significativas en los costos del ACV entre los servicios de salud de América Latina, influenciadas por variaciones en la accesibilidad al tratamiento, los resultados de los pacientes y los perfiles de riesgo clínico. Estos hallazgos ofrecen perspectivas esenciales para la formulación de decisiones políticas de salud que mejoren la sostenibilidad a largo plazo de la atención del ACV en la región.

Financiamiento: El proyecto recibió financiamiento de la World Stroke Organization y Boehringer Ingelheim (BI) IS 0135-0352.
