# Supplementary material for: Cost evaluation of acute ischemic stroke in Latin America: a multicentric study
Source: Lancet Reg Health Am. 2024 Dec 6;41:100959. doi: 10.1016/j.lana.2024.100959 (PMC11665535; doi:10.1016/j.lana.2024.100959)
Supplement: Translated Summary_POR [file mmc4.pdf]

Editorial disclaimer: *This translation in Portuguese was submitted by the authors and we reproduce it as supplied. It has not been peer-reviewed. Our editorial processes have only been applied to the original abstract in English, which should serve as a reference for this manuscript.*

Resumo

Contexto: A literatura atual ressalta uma lacuna significativa na disponibilidade de dados precisos sobre os custos do acidente vascular cerebral (AVC) na América Latina. Este estudo tem como propósito mensurar os custos reais associados ao manejo do AVC isquêmico agudo na região, utilizando a metodologia Time-Driven Activity-Based Costing (TDABC). Os resultados obtidos visam fornecer uma base robusta para a implementação de estratégias de atenção à saúde fundamentadas no conceito de valor (VBHC) no contexto latino-americano.

Métodos: Realizou-se um estudo observacional, multicêntrico e internacional que avaliou os custos diretos e os desfechos clínicos de pacientes hospitalizados por AVC isquêmico agudo, no período de dezembro de 2021 a dezembro de 2022. Foram analisados dados provenientes de centros especializados em AVC na Argentina, Brasil, Chile, Colômbia, Costa Rica, México, Peru e Uruguai. Os custos foram estratificados por país. Consideraram-se fatores como desfechos favoráveis, definidos pela escala modificada de Rankin (mRS 0-2), níveis de risco clínico e modalidades de tratamento empregadas. Para avaliar a relação entre as variáveis clínicas e o custo total por paciente, utilizaram-se modelos de Generalized Estimating Equations (GEE).

Resultados: Um total de 1.106 pacientes foi incluído no estudo. Desses, 74% receberam tratamento médico conservador, 18% foram submetidos à trombólise intravenosa (IVT), 4% à trombectomia mecânica (MT e 3% receberam terapia combinada com trombólise IVT e MT. O custo médio por paciente foi de I$ 12.203 (DP: I$ 15.055), com 49% apresentando desfecho funcional favorável. Quando comparada ao tratamento médico isolado, a MT resultou em custos 3,1 vezes superiores, com um incremento de I$ 20.418 por paciente ($p < 0,0001$). Em todos os países, os custos apresentaram aumento proporcional ao nível de risco clínico dos pacientes e às modalidades terapêuticas empregadas, sendo o tempo de internação hospitalar identificado como o principal determinante de custos.

Interpretação: Este estudo evidenciou uma significativa variabilidade nos custos associados ao AVC entre os sistemas de saúde da América Latina, determinada por diferenças na acessibilidade aos tratamentos, nos desfechos clínicos dos pacientes e nos perfis de risco. Os resultados apresentados fornecem subsídios para o desenvolvimento de políticas públicas de saúde que promovam a sustentabilidade a longo prazo na assistência ao AVC na região.

Financiamento: O projeto recebeu financiamento da World Stroke Organization e Boehringer Ingelheim (BI) IS 0135-0352.
